# Slow cortical potential neurofeedback and self-management training in outpatient care for children with ADHD: study protocol and first preliminary results of a randomized controlled trial

**DOI:** 10.3389/fnhum.2014.00943

**Published:** 2014-11-26

**Authors:** Hanna Christiansen, Verena Reh, Martin H. Schmidt, Winfried Rief

**Affiliations:** ^1^Department of Psychology, Child and Adolescent Psychology, Philipps-University MarburgMarburg, Germany; ^2^Department of Psychology, Clinical Psychology, Philipps-University MarburgMarburg, Germany

**Keywords:** ADHD, neurofeedback, self-management, slow cortical potential training, behavior therapy, effectiveness

## Abstract

**Background**: Treatment for children with attention deficit/hyperactivity disorder (ADHD) today is predominantly pharmacological. While it is the most common treatment, it might not always be the most appropriate one. Moreover, long term effects remain unclear. Behavior therapy (BT) and non-pharmacological treatments such as neurofeedback (NF) are promising alternatives, though there are no routine outpatient care/effectiveness studies yet that have included children with medication or changes in medication.

**Methods/design**: This paper presents the protocol of a randomized controlled trial to compare the effectiveness of a Slow Cortical Potential (SCP) NF protocol with self-management (SM) in a high frequent outpatient care setting. Both groups (NF/SM) receive a total of 30 high frequent therapy sessions. Additionally, 6 sessions are reserved for comorbid problems. The primary outcome measure is the reduction of ADHD core symptoms according to parent and teacher ratings.

**Preliminary Results**: Untill now 58 children were included in the study (48 males), with a mean age of 8.42 (1.34) years, and a mean IQ of 110 (13.37). Conners-3 parent and teacher ratings were used to estimate core symptom change. Since the study is still ongoing, and children are in different study stages, pre-post and follow-up results are not yet available for all children included. Preliminary results suggest overall good pre-post effects, though. For parent and teacher ratings an ANOVA with repeated measures yielded overall satisfying pre-post effects (*η*^2^ 0.175–0.513). Differences between groups (NF vs. SM) could not yet be established (*p* = 0.81).

**Discussion**: This is the first randomized controlled trial to test the effectiveness of a NF protocol in a high frequent outpatient care setting that does not exclude children on or with changes in medication. First preliminary results show positive effects. The rationale for the trial, the design, and the strengths and limitations of the study are discussed.

**Trial registration**: This trial is registered in www.clinicaltrials.gov as NCT01879644.

## Background

For children with Attention Deficit/Hyperactivity Disorder (ADHD) the European guidelines recommend a multimodal treatment (Graham et al., 2011), as well as the new German guidelines that recommend a treatment with medication only if other treatments are not effective.^1^ While this is recommended, this seems not be the reality in clinical practice. The treatment for children with ADHD today is predominantly pharmacological, with increasing prescription rates for psycho-stimulants (Barbaresi et al., 2002; Dalsgaard et al., 2013; Steinhausen and Bisgaard, 2014). it is the most common treatment and with respect to short terms the most effective one for the majority of children with ADHD (Van der Oord et al., 2008), it might not always be the most appropriate one, due to possible non-response, side-effects, or parental preferences (Lofthouse et al., 2012). Moreover, long term effects remain unclear. About one third of the children treated with stimulants does not respond (Du Paul et al., 1998; Monastra et al., 2005; Lofthouse et al., 2012), adverse medication side effects such as insomnia, and decreased appetite are often reported (U.S. Department of Health and Human Services, 1999; Schachter et al., 2001; Graham et al., 2011), and improvement often seems not to be maintained after treatment discontinuation (Swanson et al., 2001; Abikoff et al., 2004a,b; Molina et al., 2009). Of the children treated with psycho-stimulants, 44–75% do not satisfactorily profit from this treatment in long-term follow-up (MTA Cooperative Group, 1999; Swanson et al., 2001; Molina et al., 2009; Nieweg, 2010), and protective long-term effects, i.e., on substance abuse (Molina et al., 2009, 2013), or on academic achievement, social and interpersonal skills could not consistently be established (Whalen and Henker, 1991; Greenhill et al., 1999; Molina et al., 2009; van de Loo-Neus et al., 2011; Mrug et al., 2012). Accordingly, some families are hesitant about medication treatment (Visser and Lesesne, 2003; Berger et al., 2008), and treatment alternatives are warranted.

Behavior therapy (BT) and non-pharmacological treatments such as neurofeedback (NF) are promising and supposedly side effect free alternatives (Molina et al., 2009; Moriyama et al., 2012). Evidence suggests positive short-term effects for different NF protocols (Arns et al., 2009; Lofthouse et al., 2012), and there is also some, though sparse evidence for long-term effects (Arns et al., 2009; Lofthouse et al., 2012). While the efficacy as well as the need for these approaches are still discussed controversely (Jensen et al., 2007; Swanson et al., 2008; Fabiano et al., 2009), recent quantitative reviews and meta-analyses have shed light on the efficacy of non-pharmacological treatments for ADHD (Van der Oord et al., 2008; Arns et al., 2009; Fabiano et al., 2009; Lofthouse et al., 2012; Moriyama et al., 2012; Sonuga-Barke et al., 2013; Hodgson et al., 2014). Overall, those reviews and meta-analyses report robust medium to large effect sizes for non-pharmacological interventions on ADHD (Fabiano et al., 2009; Hodgson et al., 2014) as well as for NF protocols (Arns et al., 2009, 2014; Lofthouse et al., 2012; Moriyama et al., 2012; Hodgson et al., 2014; Liew, 2014). The meta-analysis by Sonuga-Barke et al. (2013) differentiates findings for NF and behavioral interventions, demonstrating larger and significant effects by raters closest to the therapeutic setting, but diminishing and non-significant effects for both interventions when probably blinded assessment (i.e., teacher ratings) was employed. Since blinded assessment was overall rare in the studies included, and reduced the already small numbers of studies subjected to meta-analysis further (from *k* = 8 NF studies to *k* = 4 with probably blinded assessment; and from *k* = 9 behavioral intervention studies to *k* = 5 with probably blinded assessment), those results should be interpreted with respect to this. More studies with higher quality and more objective outcome measures are thus warranted, though subjective improvements of parents and children are not unimportant, since an association between a positive parent-child interaction and a better outcome has been observed previously (Schachar et al., 1987; Taylor et al., 1991, 1996; Tully et al., 2004; Drabick et al., 2006; Christiansen et al., 2010).

As Lofthouse et al. (2012, p. 366) admit, blinding in psychotherapy studies is harder compared to medication studies, since knowledge of the treatment is required for a therapist and makes a placebo condition virtually impossible (see also Zuberer et al. in this Frontiers Research Topic). Nevertheless, two pilot-studies report on EEG NF double-blind randomized placebo controlled trials. Both demonstrated feasibility, but no differences between the active and placebo condition, yet (Lansbergen et al., 2011; Arnold et al., 2012). The eight existing studies using triple blinding in NF protocols are also inconclusive. Four of them report significant positive effects of medium to large size (DeBeus, 2006; Leins et al., 2006; Picard et al., 2006; DeBeus and Kaiser, 2011), whereas the four more recent ones report negative results (Logemann et al., 2010; Perreau-Linck et al., 2010; Lansbergen et al., 2011; Arnold et al., 2012). Moriyama et al. (2012, p 592), criticize, that the negative findings of those four studies “might have been determined by the use of suboptimal NF, because all of these studies used very experimental protocols and in none of them, the principals of learning theory were applied to ensure that subjects were really under conditioning procedures”. This is a key element, though, since in NF protocols operant conditioning procedures are applied to help participants learn to gain self-control over EEG patterns that are associated with attentional processes (Heinrich et al., 2007; Gevensleben et al., 2009b). Conditioning failures will thus be related to negative outcomes, since core principals of the therapy are then in question (for the combination of NF and behavioral therapeutic aspects see also Vollebregt et al. in this Frontiers Research Topic).

One study compared a NF slow cortical potential (SCP) protocol with BT (Drechsler et al., 2007). In the study, NF SCP in a single setting (20 high frequent 90 min sessions in 2 weeks with a further 5 weekly/twice weekly 90 min sessions after a 5 week break) is compared with a group therapy (total of 15 ninety minute sessions weekly to twice weekly) based on behavioral interventions such as self-management (SM) and parent training, demonstrating more pronounced treatment effects for the NF SCP group. But the total number of sessions (NF SCP: 25 vs. BT: 15), the setting (NF SCP: single vs. BT: group), frequency (NF SCP: dayly vs. BT: weekly/twice weekly), and duration of the two interventions are not comparable, hampering conclusions with respect to the efficacy of the interventions. A more recent study by Garcia et al. (see this Frontiers Research Topic) compares 57 children with ADHD that were randomly assigned to three different treatments: NF theta/beta training sessions, methylphenidate treatment, and BT. Their results reveal specific changes in EEG variables, specifically related to NF theta/beta training; results on ADHD symptoms are not reported, yet.

## Aims of the trial

The aim of the present study is to establish whether a NF protocol under outpatient care conditions is at least as effective as an approved and established behavioral treatment (SM), as results in the Drechsler et al. (2007) study suggest. The current study is designed to compare a SCP NF training with a behavioral SM training (SM: Lauth and Schlottke, 2009). To date, NF is not yet approved as a psychotherapeutic intervention by health care providers in Germany, and to our knowledge there is no effectiveness study investigating the feasibility and effects of NF under regular outpatient care conditions.

We are thus interested in whether NF (SCP training) is a true treatment alternative to behavioral interventions that are approved by health care providers. Since the studies so far were experimental ones establishing effects of NF protocols that did not allow medication or changes in medication, this question is not answered, yet.

The primary research question is:

1. Is a SCP NF protocol under outpatient care conditions at least as effective as an approved and established behavioral treatment (SM) at the end of treatment, and at six and twelve months follow-up?

Further examination of secondary research questions

1. EEG-patterns:
  Does NF result in specific changes of EEG patterns compared to SM? Are there specific associations between neuroregulation skills and clinical outcomes?
2. Child outcomes:
  Do both treatments (NF and SM) improve children’s executive functions, quality of life, self-concept and school grades? And is treatment response in both treatments moderated by children’s perceived social support?
3. Parent outcomes:
  Do both treatments (NF and SM) improve parenting skills, parental perceived social support and expressed emotion (EE)? Does the parent group with additional social support (PE + SU) show enhanced social support after treatment and more positivity and warmth towards the child compared to the group with PE only? Is this moderated by comorbidity?

## Methods

### Participants

#### Inclusion criteria

The study is performed with children either newly diagnosed with ADHD or with verified diagnoses. Participants are children referred for ADHD treatment either by their parents, pediatricians, or psychiatrists. To be eligible for the study, the children have to meet the following inclusion criteria: aged seven to eleven, full command of the German language, current DSM-IV diagnosis of ADHD (either combined, predominantly inattentive or predominantly hyperactive/impulsive subtype), IQ ≥80 (short version of the WISC; information, picture arrangement, similarities and block-design; Sattler, 2008, p. 186). Children with comorbid disorders are not excluded from the study, and behavioral treatment of comorbid conditions is included in the treatment plan. The rational for this is based on the effectiveness design of the study. The majority of the children with ADHD presents with comorbid disorders (Kadesjö et al., 2003; Willcutt et al., 2005; Gadow et al., 2006; Jakobson and Kikas, 2007; Anney et al., 2008; Semrud-Clikeman and Bledsoe, 2011; Stein et al., 2011; Vakil et al., 2012), and parents and children seeking help in our outpatient clinic request treatment of all impairing problems, and not just ADHD (please refert to the preliminary result section for information on comorbidities). The children under stimulant medication are also not excluded from the study, but dose and possible changes will be recorded.

#### Exclusion criteria

Children with symptoms of inattention, hyperactivity or impulsivity due to other medical reasons such as hyperthyreosis, autism, epilepsy, brain disorders and any genetic or medical disorder associated with externalizing behavior.

### Design and procedure

#### Recruitment and consent

The Psychotherapeutic Outpatient Clinic of the Department of Psychology, Clinical Psychology, at the University of Marburg treats children, adolescents and adults with psychological disorders. Patients can refer themselves or are referred by their pediatricians, psychiatrists, or general practitioners. Parents and children interested in the study are sent a full study description with separate information for parents, teachers, and children, and Conners-3 questionnaires for parents and teachers as well as questions on demographics and therapy expectations. Screen positive patients are invited for a semi-structured diagnostic interview (Kiddie-Sads-Present and Lifetime Version; K-SADS-PL; Kaufman et al., 1996) with a licensed child and adolescent psychotherapist to assess ADHD and possible comorbid disorders. If ADHD is diagnosed, the patient and his/her parents are informed about the treatment options and receive oral information based on the written information already sent out to the families. If the child fulfills diagnostic criteria and the family wants to participate in the study, informed consent is signed by the parents and their children, and further diagnostic assessments are scheduled.

#### Randomization and treatment allocation

The children are randomized to receive either NF or SM training. Parents of children are randomized to parent training groups with either psychoeducation only (PE), or PE enhanced with additional social support (PE+SU). Treatment allocation is performed by computer programming stratified for gender and stimulant medication. In this way, we aim to ensure that trial arms are balanced with respect to the baseline characteristics gender and use of ADHD medication. Patients, parents, therapists, and investigators were not blinded for the treatment allocation. Teachers are blind with respect to treatment allocation.

#### Procedure

Both the NF and SM interventions are manualized, equal in setting (single), duration, frequency, parental involvement, and supporting token economies (for details on this please refer to treatment protocols of this article). The rationale for those treatment parameters is based on the results of the available meta-analyses (Arns et al., 2009; Esser and Blank, 2011; Zwi et al., 2011; Lofthouse et al., 2012; Moriyama et al., 2012; Sonuga-Barke et al., 2013; Hodgson et al., 2014). The SM training is approved and refunded by insurance providers in Germany for ADHD therapy (for the efficacy of SM trainings for ADHD see the reviews by Saile, 1996; Fabiano et al., 2009); NF is only refunded in health care settings that also do research in the field. Since our department is a university one, it is possible to get NF training refunded by in such a setting.

**Figure 1:**
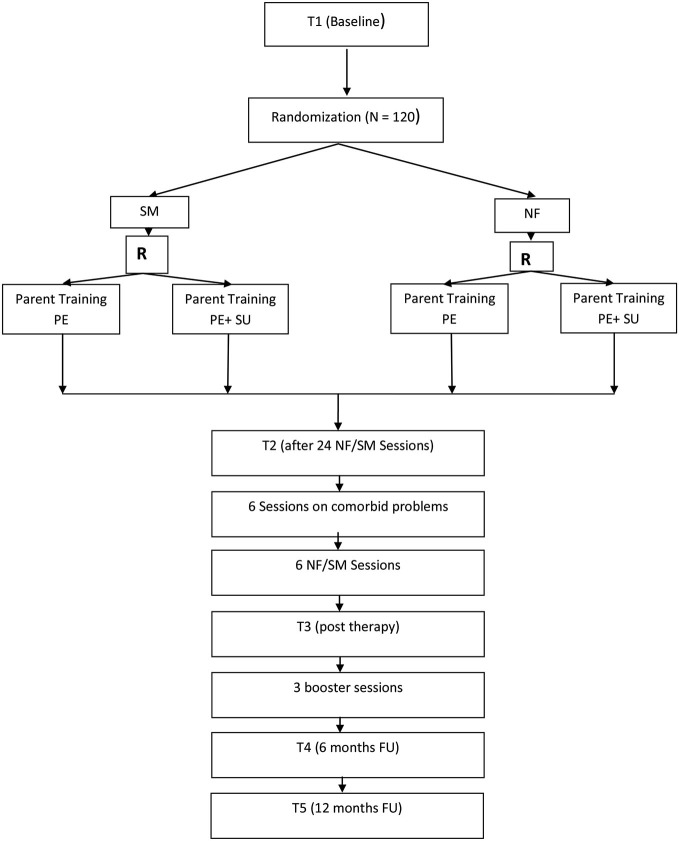
**Flow-chart of the study**. **R** = Randomization of parents to either PE or PE+SU.

Figure 1 provides an overview of the trial flow. After informed consent and baseline assessment (T1), a diagnostic assessment of ADHD and possible comorbid disorders with the K-SADS-PL follows. During T1 all primary and secondary outcome measures, neuropsychological tests, and quantitative EEGs are scheduled. Children are off medication 48 h prior to all diagnostic assessments to not distort results due to treatment. The same procedure is applied for all further assessments. After confirmation of ADHD diagnosis, randomization takes place. The children in both groups then receive 24 high frequent therapy sessions (NF or SM) over twelve weeks with up to three 1 h sessions per week, since such an intensive training has proved to be highly effective (Strehl et al., 2011). After the first twelve sessions in 4 weeks, there is a 1 week break followed by the next 4 weeks with high frequent training. After 24 sessions only Conners-3 parent ratings are used for T2 assessment and there is another break for 1 week. Additionally for both groups 6 individualized BT sessions are reserved for comorbid problems after T2 assessment. Depending on the disorder and treatment selected, those sessions might be high frequent or scheduled only weekly. After the comorbid sessions, six high frequent NF or SM follow that end the therapy. T3 assesses post-treatment effects (all primary and secondary outcomes). Five months after end of treatment, all children are offered three booster training sessions (either NF or SM according to allocation). Six (T4) and twelve months (T5) after treatment termination follow-up assessments with all primary and secondary outcome measures are scheduled. Parent groups are accompanying children’s therapy. Table 1 gives a detailed overview of the treatment plan.

**Table 1:** **Overview of all assessment and treatment sessions of the study**.

**Session**	**Content**	**Duration**
1	Outpatient assessment and informed consent; information on study participation	100 min
	**Diagnostic assessment**	
2–4	Pre-assessments of all primary and secondary outcome measures (T1)	50 min per session
	**Randomization of children and parents**	
5	Feedback of test results	50 min
6	Psycho-education children	50 min
	**Intervention**
	**Accompanying Parent Training**	
7–18	***Block I:***	50 min per session
	High frequent NF/SM treatment over 4 weeks (12 sessions)
	***Break (1 week)***
19–30	***Block II:***	50 min per session
	High frequent NF/SM treatment over 4 weeks (12 sessions)
	Break (1 week) and T2 assessment*
31–36	***Block III—part 1:***	50 min per session
	Behavior therapy of comorbid problems (6 sessions)
37–42	***Block III—part 2:***	50 min per session
	High frequent NF/SM treatment (6 sessions)
43	**Post assessment (T3)**	**50 min**
	**Follow-up assessment and booster sessions**
44–46	3 Booster sessions ca. 5 months after treatment discontinuation	50 min per session
47	6-months follow-up (T4)	50 min
48	12-months follow-up (T5)	50 min

*T2 assessment: only Conners 3© parent rating scales.

### Treatment protocols

#### Neurofeedback

Before treatment all children receive standardized PE on ADHD (Lauth and Schlottke, 2009). For NF training in this study we use the Thera Prax^®^ (NeuroConn©) NF system. It offers several different feedback animations and the option to upload pictures which keeps the training diversified and motivates children. During training sessions, the children take seat in a comfortable chair with a head- and armrest in front of a computer screen and are introduced to the training as kind of a computer game that helps them learn to modulate their brain activity.

We use the feedback protocol of SCP training that has been incorporated in many NF studies (Strehl et al., 2006; Heinrich et al., 2007; Gevensleben et al., 2009a,b, 2010; Arns et al., 2014). The children’s task is to generate negative and positive SCPs by getting into an attentive (negativity trials) or a relaxed state (positivity trials). The aim of the training is to steer a moving object (i.e., an airplane, a fish, a spaceship) that appears on the screen in front of them in the requested direction (arrow upwards indicates negativity trial; arrow downwards indicates positivity trial). The children can choose a training object at the beginning of each therapy session. In the transfer trials, the children do not see the object, but only the direction of the arrow. The children are instructed to sit as still as possible during the training, to avoid laughing and talking, but to concentrate on the screen in front of her/him. No specific instruction is given to the children on how to succeed in negativity or positivity trials, but just to be attentive to feedback and to find the most effective mental strategy to steer the object into the requested direction. As there is no unique strategy for NF training, the children are given examples that have been successful for some children (i.e., negativity trials: “Think of something you find exciting like sitting in a race car or standing on a diving board”; positivity trials: “Those strategies are used in situations requiring relaxation. Think of something you find calming and pacifying like listening to soothing music”). After a successful trial a sun appears on the screen (reinforcement). Additionally, a token plan is used that enables the children to earn up to 5 tokens per session if they stay attentive during the whole session. A full token plan of 15 tokens (every third session) can be exchanged into small rewards by parents that are agreed upon at the beginning of the training together with the parents and the child.

**Figure 2:**
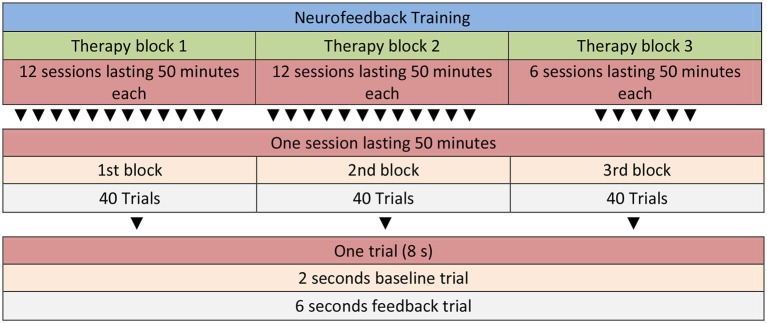
**Neurofeedback Protocol with three therapy blocks and a total of 30 sessions**.

Participants in the NF condition receive a total of 30 sessions of SCP training. Each therapy session consists of three runs. One run consists of 40 trials (8 min) resulting in a total of 24 min NF training per session (see Figure 2 for details). A trial lasts for 8 s (2 s baseline period, 6 s feedback period). Inter-trial interval is set to 5 ± 1 s. Between each of the three runs there is a short break of several minutes which can be used by the therapist to motivate and praise the child and to talk about problems and use of strategies (i.e., “What was your strategy for negativity/positivity trials?”, “How did it work?”, “What else could you try as a negativity/positivity trial strategy?”). The last 10 min of each session are reserved for joint play which is an important aspect of motivating the child and strengthening the therapeutic relationship. Feedback is calculated from the vertex (Cz) and is referenced against both mastoids (bandwidth 0.01–30 Hz, sampling rate: 256 Hz), and vertical as well as horizontal eye movements are corrected online with electrodes placed above and below the left eye, and electrodes on the right and left side of the face (4 electrooculography channels, EGO; for details of the protocol see Strehl, 2009). Ocular artifact removal is possible with DC-EEG as described in Schlegelmilch et al. (2004). The ratio of negativity to positivity is set to 1:1, and negativity/positivity trials are presented in random order. All sessions start with no threshold, but if the child has a hit rate (correct responses) of ≥70%, thresholds are introduced automatically. Those start with an initial 5% threshold, and are followed in steps of 5% if the child continuous to score ≥70%.

The first two runs in every therapy session include no transfer trials, i.e., the child gets continuous visual feedback, whereas the third run is set to 100% transfer (for this rationale see also Vollebregt et al. in this Frontiers Research Topic). Although no continuous feedback is given on transfer trials, a sun appears at the end of the trials to provide information about success (reinforcement). Transfer trials are thought to support generalization to everyday life situations (Strehl et al., 2011). Additionally, starting with the second therapy block (12th session) children are asked to practice their mental strategies in specific situations at home or school for about 10 min each day. Together with the therapist children identify situations that require attention control (i.e., negativity strategies: before starting to do homework; positivity strategies: at night before going to bed). The children are asked to document practicing by keeping a log which is controlled by the therapist at the beginning of a session (see also Vollebregt et al. in this Frontiers Research Topic). If the child practices each day between sessions he/she can earn an extra token.

In the first and second therapy block the children complete twelve NF sessions each. After those two blocks (with a 1 week break to relieve families in between; see also Strehl et al., 2011) a block reserved for the treatment of comorbid problems follows (total of six sessions). In this block individual BT is applied for the specific comorbid conditions. Treatment of those is based on published intervention manuals. Then a final block with six NF sessions follows that ends the therapy. In this block, the ratio of negativity to positivity trials is set to 3:1.

#### Self-management

Before treatment all children receive standardized PE as described in the NF section above. The SM training addresses children aged 6–12 years (Lauth and Schlottke, 2009), and is based on the self-instruction training as described in Meichenbaum and Goodman (1971). Goal of the training is to enhance child development in the domains behavior regulation, planning, organization, and self-reflection. A meta-analysis with *k* = 11 studies provides empirical evidence of the training (Lauth and Schlottke, 2009; for the general efficacy of such treatments see the reviews by Saile, 1996; Fabiano et al., 2009). In the first therapy block the basic training (12 sessions) is completed. This consists of the sessions (1) precise looking; (2) precise describing; (3) precise listening; (4) precise listening and re-telling; (5) precise account of perceptions; (6) introduction of the stop-signal; (7) self-contained stopping and checking; (8) accompanying checking procedures; (9) transfer of checking techniques to school contexts; (10) self-instruction with the stop signal; (11) self-instruction with difficult tasks; (12) self-instruction under distraction. After 1 week break the second therapy block follows with the strategy training: (1) basic skills; (2) signal cards; (3) thinking loud; (4) flexible use of signal cards; (5) adaptation of learned strategies for new tasks; (6) cross-linking strategies; (7) discover systematic principles; (8) adaptation of learned strategies for complex tasks; (9) solving abstract problems; (10) organizing learning strategies; (11) development of strategies for complex school tasks; (12) application of strategies for complex school tasks.

Each session (except the first one) begins with the recapitulation of the last session and an introduction into the topic of the session (10 min). This is followed by modeling the behavior that the topic of the session requires (10 min) and the child trains this for 20 min. The last 10 min are reserved for joint play to motivate the child and to build a good therapeutic relationship. A token plan is installed together with the child and the parents. As in the NF group, the children can earn up to 5 tokens per session if they stay attentive during the training unit. A full token plan of 15 tokens (every third session) can be exchanged into small rewards by parents.

To keep both therapy groups balanced, quantity of homework is kept identical. Starting in the second therapy block, the children in the SM group are asked to practice self-instruction with the stop signal during homework each day for 10 min. As in the NF group, practicing is documented in a log which is controlled by the therapist at the beginning of a session and the children can also earn an extra token for doing the homework. Differing from the NF protocol, the children do not practice relaxation, since this is not part of the SM training.

#### Treatment of comorbid disorders

Depending on the comorbid disorder of the child, manualized behavior treatments are applied. The most common comorbid disorder in our sample so far is Oppositional Defiant Disorder (ODD). The principles to reduce such problem behaviors are based on the training manuals used in the parent PE (see below). In addition, after PE with the child token economies are also introduced to reduce ODD symptoms. Other disorders such as Tic Disorder, Enuresis, Sibling Rivalry Disorder are also treated with BT, i.e., apart from token economies, habit reversal training, alarm therapy, one to one quality time with the child (Esser, 2011). For children without comorbid disorders, those sessions are used to resolve other conflicts that have an impairing quality, i.e., children without many friends receive social competence training.

#### Parent training

##### Psycho-education only (PE)

Since ADHD does not only affect the child, but is specifically characterized by impairments at home and school, the parent training supports the parents and the transfer into the home setting. It is part of the training by Lauth and Schlottke (2009). The core element is PE with respect to ADHD, as well as development of strategies that effectively support the child. Topics are: (1) information on ADHD; (2) handling problem behavior I and introduction of token economies; (3) joint parent-child play; (4) handling problem behavior II and introduction of timeout and 1-2-3-Magic (Phelan, 2003); (5) handling problem behavior in public. Between sessions, parents have to complete homework, training the strategies in everyday life. This homework is discussed in the following session. Therapists are the same as in the children’s intervention. A meta-analysis supports parent trainings as this one for ADHD (Bachmann et al., 2008; Zwi et al., 2011). A total of five parent group sessions lasting 100 min each are scheduled accompanying children’s therapy.

##### PE + social support (SU)

Parents in this group receive PE as described above, with additional components on social support, based on network oriented interventions (Röhrle et al., 1998), since a study has shown positive effects of social support on parental EEs, with parents with high social support showing more positivity and warmth towards their children, that was related to reduced comorbid oppositionality (Christiansen et al., 2010). Parents are trained with network analyses to identify supporting social networks, and to possibly activate them. If non-supportive network characteristics are identified, modification is supported. Between sessions, parents have to complete homework as in the PE only group and additionally on individual social network analyses. For this they have to think of ways of how to activate positive social support. A total of five parent group sessions lasting 100 min each are scheduled accompanying the children’s therapy.

### Therapists

All therapists are trained in both NF, SM, PE, and PE+SU and all therapists deliver all trainings based on intervention manuals. All interventions for comorbid conditions are also based on published intervention manuals. All therapists are therapists in training and receive regular supervision (every fourth session) by a licensed supervisor with more than 5 years experience in BT for the duration of the trial. To control implementation and fidelity, all sessions are videotaped and analyzed in the supervision sessions.

### Measurements

With the exception of parent and teacher questionnaires, all assessments are conducted in face-to-face contacts. Apart from the primary ADHD outcomes (change in psychopathology from baseline to post therapy) we are also interested in therapy related quantitative EEG patterns of response to NF/SM. In the existing NF studies, changes in EEG patterns have been reported after therapy (e. g.: Monastra et al., 2005; Heinrich et al., 2007; Gevensleben et al., 2009a; Arns et al., 2014; see also Garcia et al. in this Frontiers Research Topic). But, differing from the homogeneous results for parent and teacher ratings (primary outcomes), this change proved to be heterogeneous according to meta-analysis (Nestoriuc et al., 2011). Possible changes in EEG patterns before and after therapy will thus be compared for both the NF and SM group to establish whether changes are specific for the NF group.

#### Selection and diagnostic measurements

1. Screening for the presence of ADHD symptoms is performed with the Conners 3^®^ parent and teacher ratings (German version: Lidzba et al., 2013). Clinical impairment is established with T-scores of ≥60.
2. The DSM-IV diagnosis of ADHD and possible comorbid disorders is based on the semi-structured diagnostic interview Kiddie-Sads-Present and Lifetime Version (K-SADS-PL: Kaufman et al., 1996).

The 3rd edition of the CRS (Conners 3^®^: Conners, 2008; German version: Lidzba et al., 2013) assess ADHD core symptoms as well as Learning Problems, Executive Functioning, Peer- and Family Relations, co-morbid conditions such as ODD and Conduct Disorder (CD) in children aged six (parent and teacher forms) and eight (self-report forms) to 18 years of age. The Conners 3^®^ rating scales have been translated into German, back-translated, and norms for a German-speaking sample were established (Lidzba et al., 2013). A study on cultural comparability of the German Conners 3^®^ resulted in good model-fits for confirmatory factor analyses (Christiansen et al., submitted), and cultural comparability for a large group of Germans with Turkish migration background could also be established (Schmidt et al., 2013), with satisfactory internal consistencies of the scales in both studies.

The K-SADS-PL (Kaufman et al., 1996; German adaptation Delmo et al., 2011) is a semi-structured diagnostic interview designed to assess current and past episodes of psychopathology in children and adolescents according to DSM-III-R and DSM-IV criteria. Probes and objective criteria are provided to rate individual symptoms. The interview consists of two parts. The first part is a screening interview that screens for the psychological disorders. If an item is scored with “3” (0 = no information, 1 = nonexistent, 2 = below threshold, 3 = above threshold), the full interview of this section is carried out. Diagnoses are then based on DSM criteria that are listed and scored at the end of the full interview. The K-SADS has been carefully constructed and is widely used.^2^

#### Outcome measures

##### Parent, and/or teacher ratings

1. The Conners 3^®^ parent and teacher scales are used as the measure of change of children’s ADHD symptoms (see above Conners, 2008; German adaptation Lidzba et al., 2013). The parent and teacher scales consist of 105 and 111 items respectively that are rated on a four point Likert scale with severity ratings from 0 (not at all/never) to 3 (very much/very frequently).
2. The Parental Stress Inventory (Eltern-Stress-Fragebogen (ESF); Domsch and Lohaus, 2010) assesses with 38 items the four subscales parenting stress, role restriction, social support, and partnership. Internal consistency is satisfactory (0.76–0.92) as well as re-test reliability (0.76–0.91). Convergent validity has been established with the Parenting Stress Index.
3. The Parenting Scale (PS: Arnold et al., 1993; German version: Miller, 2001) assesses parenting styles (reactions and strategies) for different problematic situations. The two subscales over-reacting and leniency have satisfactory internal consistency (0.75), as well as the total scale (0.76).
4. Start, stop and dosis of stimulant medication are monitored throughout the therapy. Since our clinic is a psychotherapeutic department, medication treatment is monitored by children’s pediatricians or child and adolescent psychiatrist. Parents report what medication is given at what time and report on titration procedures, as well as on side-effects.

##### Child ratings

5. The Qb-Test is a combined continuous performance (CPT) and activity test for children aged 6–12 years (Ulberstad, 2012), which aims to objectively assess all three core symptoms of ADHD in one test, and has been approved by the Food and Drug Administration (FDA) in 2012. While performing a standardized CPT on a computer, the movements of the participant are recorded with an infrared camera following a reflective marker attached to a headband that the participant wears while performing the test. Factorial validity for the test and the three core ADHD symptoms (inattention, hyperactivity, impulsivity) has been established (Reh et al., 2013), as well as usefulness as a potential endophenotype assessment (Reh et al., 2014).
6. The children’s test-battery of attention assessment (KITAP: Zimmermann et al., 2002) assesses different attention parameters that are administered with a computer. Psychometric properties of the KITAP have been reported in different studies (Renner and Irblich, 2007; Kaufmann et al., 2010; Röthlisberger et al., 2010; Sobeh, 2010; Dreisörner and Georgiadis, 2011; Renner et al., 2012) as well as clinical validity for seven to 10 year old children with ADHD (Drechsler et al., 2009). In our study the following subtests are included: sustained attention, Go/No-Go, and divided attention.
7. The Child and Adolescent Social Support Scale (CASSS: Malecki et al., 1999) is a 40-item multidimensional scale measuring perceived social support from four sources: parents, teachers, classmates, and friends. Frequency ratings consist of a 6-point Likert Scale from 1 (Never) to 6 (Always). Importance ratings consist of a 3-point Likert Scale ranging from 1 (Not Important) to 3 (Very Important). Each subscale corresponds to one of the sources of support (e.g., parent, teacher, classmate, and close friend) and consists of 10 items. Subscale scores are calculated by summing the frequency ratings on the 10 items on each subscale. Analyses revealed evidence of reliability, a four-factor structure (Parent, Teacher, Classmate, and Close Friend subscales), and construct validity. The CASSS co-varies with the clinically important constructs of self-concept, social skills, and behavioral indicators. There is evidence that the CASSS can be used to understand children’s and adolescents’ perceived social support (Malecki and Demaray, 2002).
8. The self-concept interview (Schöning et al., 2002) is a structured interview. Self-concept is assessed for school, family, and peer-relations. The following categories are rated and coping abilities are assessed: social interactions, perceived quality of life and self-worth. Items are formulated in a way that they do not confound with core ADHD symptoms. Internal consistency is satisfactory (range 0.70–0.83; overall 0.85).
9. The KINDL-R (Ravens-Sieberer et al., 2003) assesses health related quality of life of children and adolescents. Both parents and children rate the six dimensions physical well-being, emotional well-being, self-worth, family related well-being, peer related well-being and school related well-being. A total of 24 items is to be rated on a 5-point Likert scale from 0 = never to 4 = often. Internal consistency is satisfactory (0.85 for the total scale, all subscales ≥0.70), and the questionnaire has been used in various studies with children (Ravens-Sieberer and Bullinger, 1998a,b).
10. The Perceived Criticism Scale (PC) consists of the item “How critical is your spouse of you” to be rated on 10-point Likert scale from 0 = not at all critical to 10 = very critical indeed. Originally the item was used by Hooley (1990) to assess high EE, i.e., hostility, criticism, and emotional over-involvement, in spouses of patients with depression. With 40% variance explained, this item was the strongest predictor of relapse in a 9 months follow-up. The item has been translated into German and was adapted for children (How much does your mum/dad like you?). This version has already been successfully used with children with and without ADHD (Christiansen et al., 2010).
11. Quantitative EEGs are assessed for both NF and SM groups before therapy (T1), and post therapy (t3) as well as at six (t4) and twelve (t5) months follow-up to establish whether NF training results in changes specific for NF.

#### Primary and secondary outcome measures

The primary outcome measure is defined as the change of ADHD hyperacitivity, inattention, and impulsivity symptoms according to parent and teacher Conners 3^®^ ratings (DSM-IV subscales and Conners’-ADHD-Index; Conners, 2008; German version: Lidzba et al., 2013) at the end of the treatment (T3) compared to T1. To establish stability of effects, T3 assessments will be compared to six (T4) and 12 (T5) months follow-up assessments. T2 assessment (after the first therapy block) will be used to establish effects compared to T1 without the treatment of comorbid disorders.

Key secondary outcome measure is percentage of treatment responders (defined as a reduction of at least 30% of ADHD symptoms according to Conners-3 ratings of parents and teachers) at the end of treatment and at follow-ups. Qb-Test (Ulberstad, 2012; Reh et al., 2013), and KITAP (Zimmermann et al., 2002) scores objectively assessing core ADHD-symptoms and executive functions at follow-ups are further key secondary outcome measures at the end of treatment (T3).

Other secondary outcome measures are changes in quantitative EEG patterns as well as changes in scores of self-concept, the KINDL-R, PC, ESF, PS at the end of treatment and follow-ups.

### Statistical analyses

Data will be analyzed according to the intent-to-treat (ITT) principle, thus patients will be analyzed according to the randomization scheme. When appropriate (data missing completely at random) the method “last observation carried forward” will be applied. The treatment effects will be analyzed with multivariate repeated measure ANOVAS with the within-subject factor “time” (five levels: T1 to T5) and the between-subject factor “group” (NF vs. SM; PE vs. PE+SU). Effect sizes will be reported with *η*^2^ = 0.039 defining a small, *η*^2^ = 0.110 a medium, and *η*^2^ = 0.200 a large effect. Gender and stimulant medications are important control variables, as well as treatment response according to primary outcomes. All analyses will be performed with SPSS 20 (SPSS Inc, Chicago, Il, USA).

#### Sample size

The primary outcome is the difference in the severity of ADHD symptoms in the Conners 3© rating scales for parents and teachers between the four treatment conditions (NF+PE vs. SM+PE vs. NF+PE+SU vs. SM+PE+SU) at the end of treatment (T3) and follow-up assessments (T4 and T5). ITT analyses as described will be performed. Meta-analyses report medium to large effect-sizes for behavioral ADHD interventions (Fabiano et al., 2009; Sonuga-Barke et al., 2013) as well as for NF (Arns et al., 2009, 2014). Sonuga-Barke et al. (2013, p. 1) state that when the best probably blinded assessment is employed, effect sizes were substantially attenuated to non-significant levels for all treatments except for free fatty acid supplementation and artificial food color exclusion. On the other hand, the meta-analyses by Fabiano et al. (2009) and Arns et al. (2009) have demonstrated homogeneous and robust effects for behavioral treatments and NF protocols, so that we expect medium to large effect sizes for parent ratings of ADHD and somewhat smaller effect sizes for teacher ratings. Thus, with an assumed effect size of *f* = 0.25, a two-sided alpha of 0.05, a power of 0.80, four groups (NF+ PE/NF+PE+SU; SM+PE/SM+PE+SU) and five measurement time points, a total of 97 children needs to be included (GPower© *λ* = 18.18, critical *F* = 1.78, numerator df = 12.00, denominator df = 276, *n* = 97, power = 0.80, Pillai V = 0.17). In order to adjust for loss of power due to an anticipated dropout of 20%, 120 children will be included in the study. Since the study is quite time consuming and the follow-up assessment fairly extensive, it seems likely that not all families will follow through with the whole study.

### Ethical review and trial registration

This RCT has been reviewed and approved by the local review board of the Department of Psychology of the Philipps-University Marburg (AZ: 2010-04). It is registered at www.clinicaltrials.gov as NCT01879644.

### Preliminary results

From February 2011 till August 2014 a total of 74 children have been screened for the study so far. Of those, 69 fulfilled study entry criteria, but 11 dropped out of the study. Thus, a total of 58 children (83% boys) has completed the diagnostic study procedure (mean age 8.42 (SD 1.34), mean IQ 110 (SD 13.37); 23% on medication; 48% with comorbid diagnoses such as ODD, Tic Disorders, Enuresis, Sibling Rivalry Disorder, Separation Anxiety Disorder). Of those, *n* = 32 children have already completed the T3 and *n* = 17 the T4 assessments. Effects of parent training groups cannot be reported here, since number of participants of the four groups is overall too small for analyses (*n* < 10 per group). Thus, preliminary data is only reported on the whole sample and for a comparison of children in the NF vs. the SM group.

An ANOVA on the whole sample with repeated measures shows significant differences between T1 and T3 scores for the Conners’ parent and teacher ratings: main effect time = *F*_(1,35)_ = 17.31, *p* < 0.001, *η*^2^ = 0.331. For details for the different subscales, please refer to Table 2A.

**Table 2A:** **Conners-3 T1 and T3 scores for parent and teacher ratings: means and standard deviations (SD) for Conners-3 raw scores, *F*- and *p*-values and *η*^2^**.

**Conners-3**	**T1**	**T3**	***F*-Value**	***p*-Value**	***η*^2^**
	*N* = 32**	*N* = 32**			
ADHD index parent	11.75 (5.53)	7.13 (5.11)	*F*_(1,31)_ = 25.23	*p* < 0.001	$\eta_{p}^{2}$ = 0.449
Inattention parent	29.56 (5.97)	15.16 (5.73)	*F*_(1,31)_ = 21.79	*p* < 0.001	$\eta_{p}^{2}$ = 0.413
H/I* parent	18.78 (7.97)	14.00 (6.79)	*F*_(1,31)_ = 32.61	*p* < 0.001	$\eta_{p}^{2}$ = 0.513
ADHD index teacher	10.59 (5.14)	7.09 (5.13)	*F*_(1,31)_ = 13.74	*p* = 0.001	$\eta_{p}^{2}$ = 0.307
Inattention teacher	17.69 (4.84)	14.78 (5.58)	*F*_(1,31)_ = 8.54	*p* = 0.006	$\eta_{p}^{2}$ = 0.216
H/I* teacher	16.03 (8.91)	12.69 (7.92)	*F*_(1,31)_ = 6.55	*p* = 0.016	$\eta_{p}^{2}$ = 0.175

*H/I = Hyperactivity/Impulsivity.

**Both SM and NF together.

An ANOVA on the whole sample with repeated measures does not show significant differences between T3 and T4 scores for the Conners’ parent and teacher ratings: main effect time = *F*_(6,11)_ = 0.59, *p* = 0.73, *η*^2^ = 0.244. For details for the different subscales, please refer to Table 2B.

**Table 2B:** **Conners-3 T3 and T4 scores for parent and teacher ratings: means and standard deviations (SD) for Conners-3 raw scores, *F*- and *p*-values and *η*^2^**.

**Conners-3**	**T3**	**T4**	***F*-Value**	***p*-Value**	***η*^2^**
	*N* = 17**	*N* = 17**			
ADHD index parent	7.53 (6.23)	7.41 (5.87)	*F*_(1,16)_ = 0.013	*p* = 0.91	$\eta_{p}^{2}$ = 0.001
Inattention parent	15.41 (6.94)	14.88 (6.13)	*F*_(1,16)_ = 0.172	*p* = 0.68	$\eta_{p}^{2}$ = 0.011
H/I* Parent	14.24 (7.79)	14.06 (8.03)	*F*_(1,16)_ = 0.26	*p* = 0.87	$\eta_{p}^{2}$ = 0.002
ADHD index teacher	5.88 (5.52)	6.12 (5.48)	*F*_(1,16)_ = 0.039	*p* = 0.84	$\eta_{p}^{2}$ = 0.002
Inattention teacher	13.76 (5.82)	13.41 (5.98)	*F*_(1,16)_ = 0.064	*p* = 0.80	$\eta_{p}^{2}$ = 0.004
H/I* teacher	10.69 (6.32)	9.88 (8.08)	*F*_(1,16)_ = 0.264	*p* = 0.61	$\eta_{p}^{2}$ = 0.016

*H/I = Hyperactivity/Impulsivity.

**Both SM and NF together.

Comparing the NF and SM group in a preliminary ANOVA with repeated measures, there is a multivariate significant main effect time (*F*_(2,27)_ = 6.98, *p* = 0.004, *η*^2^ = 0.34), but a multivariate non-significant main effect group (*F*_(2,27)_ = 0.43, *p* = 0.64, *η*^2^ = 0.03), and a multivariate non-significant interaction time^*^group (*F*_(2,27)_ = 0.01, *p* = 0.81, *η*^2^ = 0.01) for the Conners ADHD-index. Table 2C shows details of the two groups. The study continues and future results with respect to the measures outlined above and to group differences will be reported based on a larger sample.

**Table 2C:** Conners-3 T1 and T3 scores for parent and teacher ratings: means and standard deviations (SD) for Conners-3 raw scores for the SM and NF group, *F*- and *p*-values and *η*^2^ for the main effects time and time*group.

**Conners-3**	**Group**	**T1**	**T3**	***F*-Value**	***p*-Value**	***η*^2^**
ADHD index parent	SM	13.13	7.00			
	*n* = 15	(4.50)	(4.45)	Time*: *F*_(1,27)_ = 23.11	*p* < 0.001	$\eta_{p}^{2}$ = 0.461
	NF	10.64	7.71	Time*Group*: *F*_(1,27)_ = 2.89	*p* = 0.101	$\eta_{p}^{2}$ = 0.097
	*n* = 14	(6.35)	(6.28)			
ADHD index teacher	SM	10.06	6.69			
	*n* = 15	(5.48)	(5.37)	Time*: *F*_(1,27)_ = 12.99	*p* = 0.001	$\eta_{p}^{2}$ = 0.325
	NF	11.71	7.43	Time*Group*: *F*_(1,27)_ = 0.273	*p* = 0.605	$\eta_{p}^{2}$ = 0.010
	*n* = 14	(4.71)	(5.04)			

*Univariate effects for the ADHD index for time and time*group for the SM and NF group.

## Discussion

In this trial, information is collected on acceptance, feasibility, and effectiveness of behavioral treatment with either NF or SM in a high frequent outpatient setting, to establish whether NF is a treatment alternative in such a setting. To collect such information is important, since the majority of studies comparing NF to other treatments are laboratory ones, making it difficult to conclude whether such a treatment will be efficacious in a naturalistic setting. Further, the majority of studies so far was done with children either without comorbidities or stimulant treatment (Arns et al., 2009; Fabiano et al., 2009), but this is not the reality of families seeking help for their children with ADHD (see introduction). So far we were able to include 58 children and their parents in the study. About half the children present with comorbid disorders and 23% are on medication. Reasons for dropout of the study varied. For some families the setting was too time consuming, other families came from far away and were able to initialize support closer to home. The majority dropped out of the study at the beginning of the treatment. Detailed results on dropouts with respect to the NF and SM group, time points and an extensive discussion of the reasons will follow when the study is completed. So far, 32 children have completed the therapy, and 17 have completed the follow-up (T4) assessment according to the study protocol. Thus, our approach to recruit a natural sample and to treat this in the described setting was feasible so far.

First preliminary results of our study show positive training effects. Children in both groups (NF and SM) improve in their psychopathology ratings according to parent and teacher Conners-3 scores over time. There is no significant difference between groups (NF and SM) in changes over time. Since we assumed that NF treatment will be at least equally effective, this assumption is met. So far, those effects are stable over time, since there is no significant change in Conners-3 scores from post treatment (T3) to 6 months after treatment (T4). Since this is an ongoing study we could only include 32 children in our preliminary analysis. Thus, those results should be perceived with caution. Results on primary and secondary outcomes with respect to our research questions (i.e., group differences, long-term effects, response rates, objective measures, changes in medication etc.; see above) and for all four groups (NF/SM, PE/PE+SU) will be presented when the full data set is available. But, if this treatment in a time limited, high frequent outpatient setting (three times a week over a period of 12 weeks) continues to be as effective as our preliminary results suggest, NF training might be an additional treatment alternative for other outpatient clinics and private practices. This would contribute to an improved patient centered care for this large group of impaired children (Christiansen and Röhrle, 2012).

The greatest challenge of the study so far is the high frequency of sessions. Today, the majority of children is involved in extracurricular activities and/or parental duties make appointments three times a week difficult. The total time frame (12 weeks) somewhat eased reservations towards participation though, especially the fairly fast positive experiences related to the treatment have proved to be very motivating for children and parents. Considering the many studies that demonstrated shortend delay reward gradients for children with ADHD, i.e., a preference for smaller but sooner rewards (Sagvolden, 2000; Kuntsi et al., 2001; Solanto et al., 2001; Sonuga-Barke, 2002, 2011; Dalen et al., 2004; Antrop et al., 2006; Hoerger and Mace, 2006; Bitsakou et al., 2009; Tripp and Wickens, 2009), this seems crucial for positive therapy effects, and indeed argues for short and frequent therapy time frames, while coming to the therapy sessions might not necessarily be perceived as rewarding.

## Limitations

A limitation of the study is the lack of blinding. Even though randomization and stratification of study participants are carefully done, treatment allocation is not blinded, as are of course neither children, nor parents and therapists. To meet this limitation, we decided to include likely objective outcome measures as key secondary outcomes, i.e., the Qb-Test and the KITAP. Both are computer based and assess the three ADHD core symptoms (Qb-Test: Reh et al., 2013), and differential markers of inattention (KITAP; Drechsler et al., 2009). Further, the probably blinded assessment in the meta-analysis by Sonuga-Barke et al. (2013) were teacher ADHD ratings and those are also part of the primary outcome in our study. Those strategies, along with an a priori power analysis and assessment of participants with and without medication have been suggested to optimize designs in NF research (Vollebregt et al., this Frontiers Research Topic).

All parents receive parent training. This in itself is an evidence based intervention (Bachmann et al., 2008; Zwi et al., 2011), and could cause confounder effects, especially since the PE part includes strategies to manage problem behavior. But, children aged seven to eleven rarely refer themselves to therapy (Kazdin, 2003), and psychotherapy effects for children are larger, when parents are involved (Esser and Blank, 2011). Thus, not to include parents would be against the state of the art, and would not respect the needs of parents and caregivers.^3^ It might be difficult to differentiate parent training effects for the two groups. But since the PE part is identical in both groups, and the SU does receive an addition on network-analyses, we do hope to be able to discriminate effects for the two different conditions in this study.

## Conclusion

Despite these challenges and limitations, we think that this study is a first step in establishing effective interventions in primary psycho-therapeutic care for parents and children seeking help for ADHD. According to our preliminary results, NF and SM accompanied by parent training seem to be effective in a high frequent outpatient setting. Since 23% of the children are on medication, NF and SM training effects seem to result in additional improvement. While the efficacy of psychological treatments for children has frequently been shown, the dissemination in routine care is still a problem to be solved.

## Author’s contributions

Hanna Christiansen designed the study and drafted the manuscript. Verena Reh and Martin H. Schmidt conduct the study and participated in the design of the study and performed the power analysis. Winfried Rief conceived of the study, and participated in its design and coordination and helped to draft the manuscript. All authors read and approved the final manuscript.

## Author’s information

Hanna Christiansen, PhD, is a professor of clinical child and adolescent psychology (chair) at the University of Marburg whose main research interests are neuropsychology and treatment of ADHD, children of mentally ill parents, and prevention of mental disorders.

Verena Reh, PhD is a clinical psychologist whose main research interests are behavioral assessment methods for ADHD, and new psychological treatment options for childhood ADHD and comorbid disorders.

Martin H. Schmidt is a psychologist (Dipl. -Psych.) and PhD student whose main research interests are assessment methods for ADHD in childhood and in adulthood, and new psychological treatment methods for children, adolescents, and adults with diagnosis of ADHD.

Winfried Rief, PhD, is a professor of clinical psychology and psychotherapy (chair) at University of Marburg, head of the outpatient clinic for psychological interventions, and head of the postgraduate training program in cognitive-BT at University of Marburg, Germany.

## Conflict of interest statement

The authors declare that the research was conducted in the absence of any commercial or financial relationships that could be construed as a potential conflict of interest.

## Footnotes

^1^http://www.awmf.org/leitlinien/detail/ll/028-019.html

^2^http://www.psychiatry.pitt.edu/research/tools-research/ksads-pl

^3^http://www.nice.org.uk/guidance/cg72 (retrieved August 18th 2014).
